# Factors that influence career progression among postdoctoral clinical academics: a scoping review of the literature

**DOI:** 10.1136/bmjopen-2016-013523

**Published:** 2016-10-21

**Authors:** Veronica Ranieri, Helen Barratt, Naomi Fulop, Geraint Rees

**Affiliations:** 1 Academic Careers Office, School of Life and Medical Sciences, University College London, London, UK; 2 Department of Applied Health Research, University College London, London, UK; 3 Health Care Organisation and Management, Department of Applied Health Research, University College London, London, UK; 4 Faculty of Life Sciences, University College London, London, UK; 5 Department of Cognitive Neurology, University College London, London, UK; 6 Academic Careers Office, University College London, London, UK

**Keywords:** MEDICAL EDUCATION & TRAINING, Early Career Clinical Academics, Early Career Clinical Academic Progression, Clinical Academic Careers

## Abstract

**Background:**

The future of academic medicine is uncertain. Concerns regarding the future availability of qualified and willing trainee clinical academics have been raised worldwide. Of significant concern is our failure to retain postdoctoral trainee clinical academics, who are likely to be our next generation of leaders in scientific discovery.

**Objectives:**

To review the literature about factors that may influence postdoctoral career progression in early career clinical academics.

**Design:**

This study employed a scoping review method. Three reviewers separately assessed whether the articles found fit the inclusion criteria.

**Data sources:**

PubMed, Scopus, Web of Science and Google Scholar (1991–2015).

**Article selection:**

The review encompassed a broad search of English language studies published anytime up to November 2015. All articles were eligible for inclusion, including research papers employing either quantitative or qualitative methods, as well as editorials and other summary articles.

**Data extraction:**

Data extracted from included publications were charted according to author(s), sample population, study design, key findings, country of origin and year of publication.

**Results:**

Our review identified 6 key influences: intrinsic motivation, work–life balance, inclusiveness, work environment, mentorship and availability of funding. It also detected significant gaps within the literature about these influences.

**Conclusions:**

Three key steps are proposed to help support postdoctoral trainee clinical academics. These focus on ensuring that researchers feel encouraged in their workplace, involved in collaborative dialogue with key stakeholders and able to access reliable information regarding their chosen career pathway. Finally, we highlight recommendations for future research.

**Strengths and limitations of this study**

- In this article, we identify six factors that may influence career progression among postdoctoral clinical academic trainees.
- Our methodological approach enabled us to include a wide range of types of literature, from empirical studies to editorials.
- Including such a broad range of literature may have introduced a risk of bias.
- The literature we sourced was predominantly North American, so may be of limited relevance to clinical academic training in other countries.

## Introduction

Concerns about the future of academic medicine and, in particular, a potential shortage of trainee clinical academics have been expressed worldwide.^1–3^ A concern of particular significance is the failure to retain postdoctoral trainee clinical academics in clinical academic careers, once they have completed doctoral studies.^4^ This is noteworthy as the sustainability of academic medicine and future improvements in clinical practice are both contingent on a continuous pipeline of researchers.^5–7^

In response to these concerns, a number of bodies around the world have invested a substantial amount of effort into improving the recruitment and retention of medical academic researchers. For instance, in 2003, an international group of medical academics, academic publishers and stakeholders came together to form a campaign to promote partnerships and global debate about how best to revitalise academic medicine.^8^ In England and Wales, this campaign preceded the roll out of a new Integrated Academic Training (IAT) programme for clinicians. The aim of this programme, overseen by the National Institute for Health Research, was to develop a clear pathway for aspiring medical academics. Trainees on this pathway receive protected research time that includes their medical training.^9^ This pathway consists of three key specialist academic training stages: a predoctoral Academic Clinical Fellowship, succeeded by a doctoral training fellowship and, finally, an Academic Clinical Lectureship.^9^

The IAT programme has yet to be formally evaluated. However, there are indications that only around a third of trainee doctors who complete a PhD within the UK progress to posts with clinical and academic responsibilities.^10–12^ These figures suggest that some postdoctoral trainee doctors experience either a change in their career preferences, having completed a PhD, or difficulties in their career progression that prevent them from pursuing academic medicine.

Examining the available literature about the experiences of early career trainee clinical academics may help us understand the factors that influence this cohort's choice to pursue or discontinue a career in academia. The aim of this review is to describe the range of motivators, facilitators and barriers experienced by this group in their career development. For the purpose of this study, we use the term ‘early career clinical academics’ to describe medically qualified postdoctoral researchers who are in a clinical academic position typically within 5–7 years of obtaining their PhD qualification. From the factors identified in this scoping review, we make a number of proposals that could help support early career trainee clinical academics, as well as highlight areas where future research is warranted.

## Methods

### Scoping review

The goal of this review was to map out the literature on the factors that influence career progression among postdoctoral clinical academics and identify areas within the research that need further clarification and emphasis. To achieve this, we undertook a scoping review as this method provides a way to synthesise a broad outline of the available evidence.^13–16^ Although systematic reviews may be viewed as the ‘gold standard’ when evaluating the evidence on a topic, they typically seek to answer specific questions, by summarising only the findings of studies with a predetermined design.^13^ In comparison, scoping reviews seek to describe the full range of literature relating to a broad area of research, including publications with a range of designs, such as editorials and systematic reviews, as well as quantitative or qualitative research articles.^13^

Arksey and O'Malley's^13^ initial framework for conducting a scoping review guided our method. This framework was chosen for its comprehensiveness and wide use in scoping reviews. This process consisted of identifying a clear research question; searching for and selecting relevant studies that aimed to answer our research question; and charting, summarising and reporting the findings of these studies. The framework does not include a quality assessment of included articles, as scoping reviews are designed to be rapid and broad in nature, as well as inclusive of all types of articles.^14–16^ Although later modifications have suggested that a quality assessment be included in scoping reviews, such assessment was not feasible for this review because no single tool exists for consistently appraising the breadth of the article types sought; qualitative, quantitative and opinion.^14^ ^16^ In line with recent enhancements to the framework, discussion between team members and separate reviewing of a proportion of abstracts and full-text papers were undertaken to ensure that relevant studies were included in the review.^14–16^ All potentially relevant articles references were uploaded onto referencing software where duplicates were accounted for a removed manually. (EndNote X5 [program]. X5 version: Thomson Reuters, 2016) Screening was conducted by VR, and a selection of included articles was reviewed by two other authors (HB and NF) to ensure that the topic they addressed adhered to our inclusion criteria.

### Search strategy

This scoping review encompassed a broad search of English language studies published anytime up to November 2015. The following search engines were chosen as they covered a broad range of journals within health sciences: Web of Science, Scopus and PubMed. Google Scholar was used as an additional search engine to identify additional studies, including those present within the grey literature. Our search terms were (‘academic medicine’ OR ‘clinical academia’ OR ‘clinician scientist’ OR ‘research physician’) AND (‘barriers’ OR ‘motivators’ OR ‘facilitators’ OR ‘predictors’) AND (‘career progression’ OR ‘career development’) within the publication. Further information pertaining to our search terms is given in online supplementary appendix 1.

10.1136/bmjopen-2016-013523.supp1 supplementary appendix

We included all English language publications that examined any influences experienced by early career clinical academics in any medical specialty. This included either the current perspectives of early career clinical academics or the retrospective viewpoints of researchers who progressed to a more senior position or left clinical academia following an early career post. In this study, early career clinical academics referred to individuals who had recently completed a PhD and in a junior research post typically lasting no more than 5–7 years since their completion date. We excluded publications if they did not include early career clinical academics within their sample or where none of the participants sampled held a PhD. The method adopted within each publication did not form part of our inclusion or exclusion criteria as studies using a range of methods were included.^13^

### Data extraction and synthesis of results

We created a spreadsheet in Microsoft Excel to collect relevant data from each paper. Data extraction was performed by VR. Data were summarised qualitatively and quantitatively. To facilitate this, we extracted data regarding the following characteristics from all included studies: author(s), sample population, study design, key findings, country of origin and year of publication. Overarching themes were identified inductively from the study findings by VR and reviewed by all authors. A narrative summary was created for each theme.

## Results

**Figure 1:**
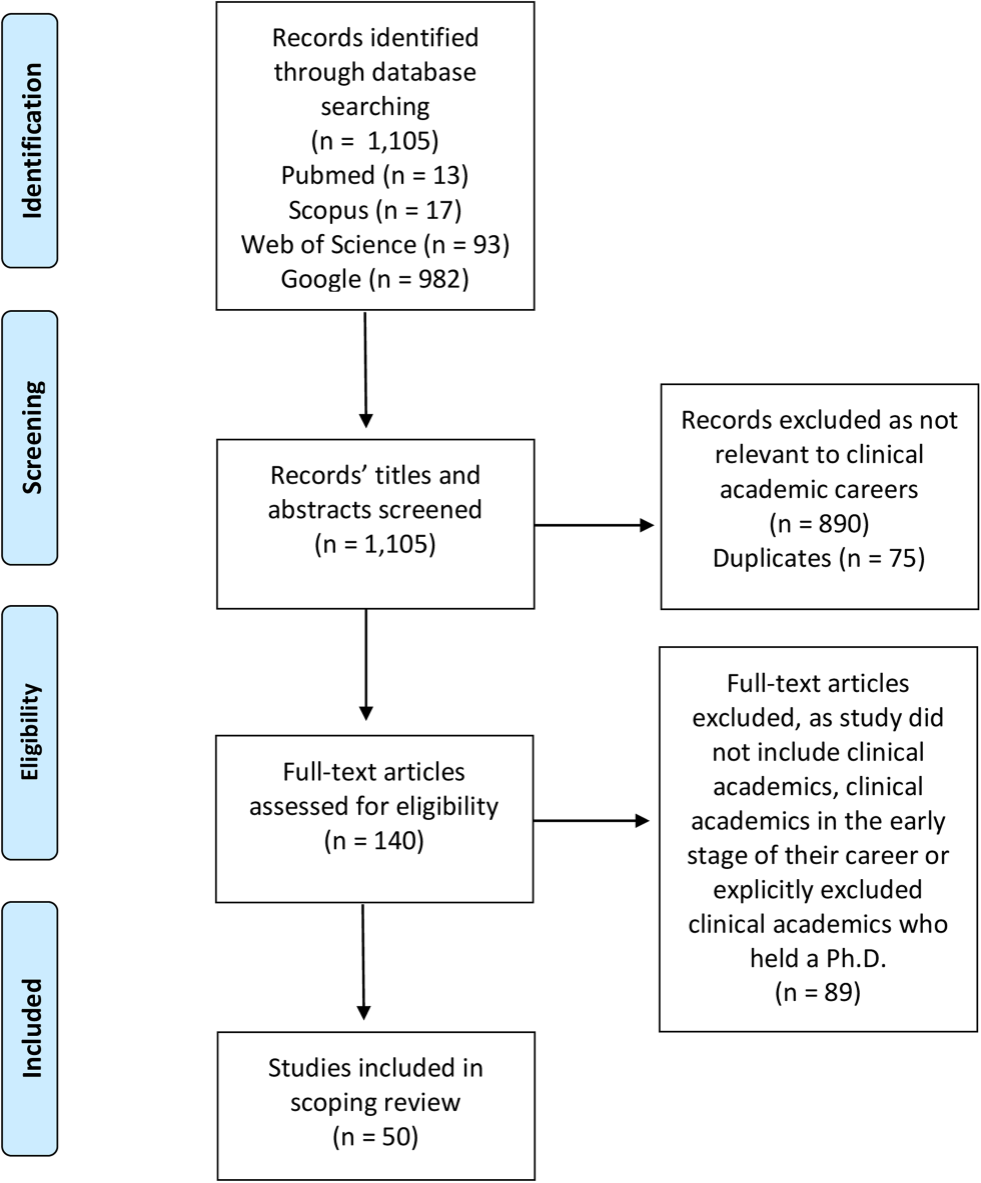
A PRISMA flow chart diagram of the scoping review process.^17^

Our database search produced 1105 potentially relevant articles. After assessing the eligibility of these by title and abstract, 890 articles were excluded as they did not meet our inclusion criteria. For example, excluded articles may have focused on different clinical academic professional groups, such as nurses, or on senior trainee clinical academics. The remaining 140 articles were then screened according to the same criteria on the basis of their full text. A total of 50 articles were included in the final review (see figure 1).

### Type of literature

Included articles were predominantly American (n=40). A further six articles were Canadian and the remaining four articles were British. The majority of included articles reported empirical research (74%, n=37). The remaining articles consisted of either editorials or commentaries. Publication dates spanned from 1991 to 2015 and papers were identified from 32 different journals. Information about the type of articles included in this review can be found in table 1. Six themes emerged on identifying and synthesising commonalities and contrasts found within the literature, as presented below.

**Table 1:** Types of publications reviewed (N=50)

Types of publications	No of publications reviewed	Examples
Editorial or commentary	9	Kubiak *et al* (2012)^18^ Kalia (2003)^19^
Empirical	34	Bucklin *et al* (2014)^20^ Steele *et al* (2013)^21^ Kalfoglou *et al*. (2002)^22^
Literature/systematic/workshop review	6	Straus *et al*. (2006)^23^
Case study	1	Lander *et al* (2010)^24^

#### Theme 1: intrinsic motivation

Intrinsic motivation, that is viewing academic medicine as rewarding and stimulating an inner interest or desire to discover, has been found to be an important factor in the literature.^25^ Early career trainee clinical academics perceived research to be highly valuable and pursued it despite uncertainty of career success.^26^ ^27^ These individuals found their role intellectually stimulating and discovery exciting.^28^ Academic medicine, to those who were intrinsically motivated, afforded them with the opportunity to engage in different roles, for example teaching.^18^ ^28^ Following rejection from academic journals and funding bodies, early career researchers who were intrinsically motivated developed resilience and persevered in their careers.^29^

#### Theme 2: work–life balance

Achieving a work–life balance in a time-constrained career is a challenge to many young academic physicians.^30–32^ Concerns regarding the ability to balance the already substantial workload of academic medicine with activities, such as childbearing and child rearing, are viewed as barriers to career development in academic medicine. Such barriers, though significant, were potentially overcome by financial stability and positive personal and social conditions in the postdoctoral researcher's life.^30^ ^33^ When the option of part-time work to meet the needs of those with more challenging work–life scenarios was unsupported by senior colleagues, a larger proportion of junior clinical academics elected to leave academic medicine.^34^

#### Theme 3: inclusiveness

A third influence which emerged was career discrimination based on gender or race. However, gender-based studies of career advancement in academic medicine have highlighted some contradictory issues.^35–37^ Recent evidence indicates that there is no difference between genders in terms of receipt of governmental funding, number of publications or h-index at an early career stage.^36^ ^38^ However, earlier studies suggested that women were awarded only 31–47% of US National Institutes of Health (NIH) career development awards between 1997 and 2008 (K08/K23).^39^ Furthermore, men outnumber women in junior clinical academic positions with over half of assistant professorships being held by men.^39^ ^40^

Nevertheless, female early career researchers perceive gender-based discrimination as a significant barrier to career development.^41^ Desire to leave clinical academia was much greater in women than men who were in a junior faculty position.^42^ ^43^ A former junior female faculty identified a number of factors that led them to leave academic medicine. Both a shortage of female role models that championed balancing work and family responsibilities (and inspired others to do the same) and low remuneration discouraged female physicians from pursuing an academic career.^44–47^ Female clinician scientists distinctly expressed doubt regarding their ability to advance and successfully integrate family life with a career in academic medicine.^19^ ^48^ In addition, a lack of role models created an unwelcoming environment for ethnic minority staff and led to an environment in which staff felt that they did not belong and left their employment.^49^ ^50^

#### Theme 4: work environment

Allied to influences present within the individual, were influences experienced externally. Three principal themes related to the individual's environment emerged from the literature: type of work environment; access to mentoring and availability of funding. Early career trainee clinical academics expressed a desire for an inclusive, respectful and flexible environment that promoted creativity and academic freedom.^20^ ^51^ ^52^ A junior faculty valued institutions that were committed to their career development. They were discouraged by institutional failure to formally recognise their dedication to teaching and ambiguity regarding their pathway to promotion.^20^ ^27^ ^53^ ^54^ Pressure to prioritise clinical duties over research and teaching was frequently cited as a negative aspect of early career clinical academics' working environment.^55^ Those who left academic medicine described their research institution as unwelcoming and individualistically competitive.^44^ ^51^ Feeling isolated from peers, sensing a lack of support and fearing retribution for open communication deterred junior trainee clinical academics from pursuing academic medicine.^20^ ^51^ Furthermore, returning to a research unfriendly residency following completion of a PhD and being appointed to a temporary research rather than tenure track position in the USA were linked to attrition from academic medicine.^20^ ^27^ ^56^

#### Theme 5: mentorship

The fifth theme we identified is mentorship, which studies suggest is often experienced to differing degrees in the workplace. Early career researchers emphasised how important a research supervisor was to their academic career advancement.^21^ ^57^ ^58^ Those who were based in an academic setting that nurtured supportive mentorship and positive role modelling tended to pursue academic medicine with greater career satisfaction and confidence.^59–61^ Optimal mentorship was marked by altruistic guidance and clarity from supervisors who encouraged junior faculty.^18^ ^52^ Effective mentors provided moral and institutional support to aid in their mentee's personal and career development.^23^ ^60^ ^62^ Such mentorship was found to be particularly critical when trying to secure independent funding and in building resilience in the face of grant or publication rejection.^29^ ^33^ Those who successfully attained a postdoctoral post attributed some of their success to mentorship that increased their level of trust in their own capabilities, promoted greater independence of thought within research and desire to remain within clinical academia.^63^ Nevertheless, to some, identifying a suitable and available mentor to guide, advise and critique their work was a significant barrier and led to attrition.^18^ ^27^ ^64^ Effective mentoring was undermined by relational shortcomings such as poor working alliance and ineffective communication and functional shortcomings linked to time constraints and lack of incentive from the mentee.^60^ ^62^

#### Theme 6: availability of funding

The availability of funding is the final key theme we identified in the literature as being crucial to early career clinical academics' career progression.^65^ Difficulties in acquiring research grant funding that allowed for protected time for research were cited as a barrier to career progression in early career researchers.^22^ ^66^ ^67^ Attaining funding was limited by a perceived loss or scarcity of funding sources.^52^ This, in turn, was attributed to a decrease in governmental investment in research and competition with full-time researchers for the same grants in Northern America.^24^ ^55^ Furthermore, financial pressures such as concerns regarding debt management and pay equity between clinical and academic faculties were perceived as demotivating.^18^ ^24^ ^64^

## Discussion

This scoping review was conducted to inform the debate about the sustainability of academic medicine. We identified six key themes in the literature, which may influence academic career progression among early career clinical academics. These themes include the role of intrinsic motivation in the development of a clinical academic career, the difficulties inherent in achieving a work–life balance in academic medicine and perceived differences in career progression according to demographic factors such as gender and ethnicity. Our review also highlights the positive influence of supportive mentorship on career advancement and the effect that the workplace environment may have on early career trainee clinical academics' motivation to remain in academia. Finally, one of the most frequently cited themes was the availability of funding and the negative implications of limited access to such funding for an early career clinical academic's career progression. In line with our search strategy, each influence can be regarded as either a facilitator or a barrier to career progression.

This scoping review is novel in that it collates and synthesises the evidence pertaining to factors or influences affecting clinical doctoral trainees' academic career decisions following the completion of their doctoral studies. Unlike previous reviews, it distinctly emphasises the completion of a doctorate as a focal entry point into a clinical academic career and focuses on the experiences of those who recently completed this milestone.^23^ Its key strength is that it describes a wide range of literature, ranging from editorials to systematic reviews that examine pertinent aspects or influences, to produce an overall narrative of the experiences of early career trainee clinical academics. While many of the research articles we found tended to provide detailed descriptions of specific influences experienced by our target population, they often failed to provide a bigger picture. Editorials, conversely, were more adept at doing so but, we found, were often not cited in research articles. These, however, carry a risk of bias as they are based on opinion rather than science. Recent articles have urged researchers to perform a quality assessment of the included literature in an effort to improve the robustness of scoping reviews.^14–16^ ^68^ As this review included a mixture of quantitative, qualitative and editorials, there was no single quality assessment measure available to us that embraced the wide range of research designs included. Nevertheless, this exercise provided us with an enriched narrative of what is known on the topic. Furthermore, this review provides details such as the sample size and study location of the included articles, so that readers can determine the generalisability of the studies.

The literature we found was important in revealing the influences experienced by trainees. However, some of the studies explored the views of junior and senior researchers and did not distinguish between the views of these two groups.^18^ ^51^ ^52^ ^62^ ^63^ These studies may, therefore, not wholly represent the views of junior researchers. Second, some of the included studies did not specify whether all early career trainee clinical academics held a PhD.^20^ ^21^ ^26^ ^29^ ^30^ ^59^ In searching specifically for published data relating to our target group, it is possible that we may have overlooked influences that are likely to affect early career clinical academics, but are only reported in the broader career literature. Additionally, in limiting our search to our specific search terms, we may have restricted the articles produced by our search, thus limiting our range of possible themes.

The themes identified from this scoping review are unlikely to apply to academic medicine alone. For example, gender inequality features in many facets of academia and, in the UK, has led to the Athena SWAN Charter, a charter designed to encourage the participation of women in higher education and research.^69^ However, highlighting and synthesising the findings of international studies may only partially shed light on the experiences of early career clinical academics in the UK. Nevertheless, drawing on our findings, we propose that there are three key actions we can take to help encourage our early career clinical academics.^12^ Ensuring that junior researchers feel integrated and supported in their work environment, for example, by improving mentoring or supervisory practices, may act as a significant first step. Including early career clinical academics in a collaborative dialogue with key stakeholders, such as universities and sponsoring bodies, may help this group feel involved in career decisions that affect them. Finally, collecting routine data on clinical academic training pathways and trainees' experiences from these universities and sponsoring bodies and regularly releasing such data to those who need it may help inform the format of future academic training for clinical researchers.

Our review highlights a number of significant gaps in our current knowledge. The literature we reviewed tended to focus on the barriers that impeded career advancement. Much less was found on the motivations to continue to pursue clinical academia. For example, although we are now familiar with the positive effects of supervisory research mentorship, we do not yet know whether different forms of mentoring affect postdoctoral trainee clinical academics' career progression. On a practical level, the next step in advancing our knowledge of the experiences of this cohort is to examine the effect of the above themes on career progression according to career grade and discipline. In doing so, we need to also ask postdoctoral trainee clinical academics about their perceptions of accessing funding and satisfaction with supervisory (or other) mentorship. Within the UK, this examination should include a formal evaluation of the IAT programme. This exercise should assess whether our junior clinical researchers perceive a clear and unobstructed trajectory into academic medicine.

Although there is limited information available to describe the experiences of trainee clinical academics, a range of theories have been set out in the broader career development literature to account for why individuals may remain in a chosen career pathway. We argue that embracing a theoretical stance when examining these perspectives may provide a better understanding of how these themes impact early career clinical academics. Incorporating such theory may help us to objectively recognise the underlying mechanisms at play in this group's career decisions.^70^ It may also help us sculpt our proposals and methods of intervention accordingly. However, no theory alone could sufficiently account for all the influences that trainee clinical academics may experience in their careers. Instead, we propose applying a systems theory framework that integrates a number of influencing factors identified in prominent theories of career development and present these as one metatheory.^71^ Using this framework, such influences are divided according to whether they are present within the individual (for instance, beliefs regarding one's ability to succeed in a chosen career path, as outlined in self-efficacy theory and social cognitive career theory), within the individual's immediate social system (eg, perceptions of disparity within the workplace that may lead to disengagement, as proposed by the psychological contract theory) or within the greater environment (such as the influence of globalisation, geography, socioeconomics and employment opportunities).^72–78^

## Conclusion

To conclude, this scoping review provides a synthesis of the influences that are experienced by early career clinical academics in their academic career progression internationally. In line with the themes found, three key steps are proposed to help support this group. In light of changes to clinical academic training in the UK, a key next step must be to evaluate whether junior trainee clinical academics perceive a clear and unobstructed future trajectory into academic medicine.
