# Supplementary material for: Factors that influence career progression among postdoctoral clinical academics: a scoping review of the literature
Source: BMJ Open. 2016 Oct 21;6(10):e013523. doi: 10.1136/bmjopen-2016-013523 (PMC5093673; doi:10.1136/bmjopen-2016-013523)
Supplement: supplementary appendix [file bmjopen-2016-013523supp_appendix.pdf]

APPENDIX 1: Searching databases

Our search string was initially created for PubMed and later extended to Web of Science, Scopus and Google Scholar databases. Trial searches were conducted to test the suitability of different search terms and search strings in returning an optimal number of results. As a consequence of this process, more restrictive terms related to funding bodies or postdoctoral schemes were avoided. Search terms were discussed and approved within team.

Search dates: 1991-2015

Our search string was (“academic medicine” OR “clinical academia” OR “clinician scientist” OR “research physician”) AND (“barriers” OR “motivators” OR “facilitators” OR “predictors”) AND (“career progression” OR “career development”).
